# An effective approach for treating unresectable hepatoblastoma in infants and children: Pre-operative transcatheter arterial chemoembolization

**DOI:** 10.3892/ol.2013.1444

**Published:** 2013-07-03

**Authors:** JING ZHANG, FEI XU, KUNSHAN CHEN, SHAOYI ZHOU, HAIBO LI, CHUANQIANG NIU, XIAOYUN TAN

**Affiliations:** 1 Department of Interventional Radiology and Vacular Anomalies, Guangzhou Women and Children’s Medical Center, Guangzhou Children’s Hospital, Guangzhou, Guangdong 510623, P.R. China; 2 Department of Respiratory Medicine, The First Affiliated Hospital, Nanchang University, Nanchang, Jiangxi 330006, P.R. China

**Keywords:** hepatoblastoma, transcatheter arterial chemoembolization, tumor volume, α-fetoprotein

## Abstract

The objective of the present study was to investigate the feasibility and efficacy of pre-operative transcatheter arterial chemoembolization (TACE) for unresectable hepatoblastoma in infants and children. A total of 24 patients (14 males and 10 females) with unresectable hepatoblastoma, aged between 26 days and 41 months, were treated with pre-operative TACE between March 2007 and March 2011. All cases were confirmed by computed tomography (CT) and liver tumor biopsy prior to TACE. Arteriography was performed and the chemoembolization mixture (pirarubicin and cisplatin emulsified in lipiodol) was injected, followed by polyvinyl alcohol (PVA). The procedure was performed one to four times depending on the patient’s response. There was a significant reduction in tumor volume associated with decreased α-fetoprotein (AFP) levels following TACE. Tumor volumes decreased by between 46.1 and 90.2%, with a mean value of 72%. The AFP levels fell by between 63.8 and 99.9%, with a mean value of 95.7%. A total of 22 cases underwent subsequent safe complete surgical resection and the remaining two patients accepted a partial resection. To evaluate the toxicity of TACE, the alanine aminotransferase (ALT), serum creatinine (Cr) and creatine kinase (CK) levels of the patients were measured to assess liver, renal and cardiac function, respectively. The results showed that no marked chemotherapeutic agent-induced toxicity occurred during TACE. It may be concluded that TACE is an effective and feasible pre-operative therapeutic approach for treating unresectable hepatoblastoma and that it may improve the resectability of bulky liver tumors.

## Introduction

Hepatoblastoma (HB) is the most common malignancy of the liver in infants and children, comprising ~1% of all pediatric cancers (1–3). It is well known that a complete surgical resection is vital to cure patients with HB. However, 50% of cases do not have this chance due to advanced and unresectable HB at the initial presentation (4–6). In this situation, it is considered that a poor patient prognosis is associated with a large tumor size, porta hepatis invasion and metastatic spread (5–7). HB has been recognized to be a highly chemosensitive tumor since the 1970s (8). Therefore, pre-operative systemic chemotherapy acts to improve the prognosis of children with HB by reducing the tumor size and increasing the feasibility of complete resection. However, the toxicity of systemic chemotherapy, including cardiac and bone marrow damage, is occasionally significant (9). In order to reduce these disadvantages, a new chemotherapeutic approach that is able to improve the survival rates of these patients is urgently required. Transcatheter arterial chemoembolization (TACE) has been attempted for the treatment of unresectable HB and may be an alternative to systemic chemotherapy (10–12). The present study analyses the use of pre-operative TACE in 24 patients with HB who were treated over the last four years at the Guangzhou Women and Children’s Medical Center (Guangzhou Children’s Hospital, Guangzhou, Guangdong, China). According to the results of these patients, the feasibility and effectiveness of pre-operative TACE for infants and children with HB was investigated.

## Materials and methods

### Patients

The present study was approved by the Ethical Committee of Guangzhou Women and Children’s Medical Center and written informed consent was obtained from all patients’ legal guardians. Between March 2007 and March 2011, 24 cases with HB were considered suitable for the present study. These cases consisted of 14 males and 10 females, with ages ranging between 26 days and 41 months, with a mean age of 14.2 months. The chief clinical manifestations of all patients were an abdominal mass, bloating and significant elevation of the serum α-fetoprotein (AFP) level. All cases were confirmed by computed tomography (CT) and live tumor biopsy prior to TACE. Tumors were considered unresectable if they were bilobar or multicenteric. According to the Children’s Cancer Study Group (CCSG) staging system (13), there were three cases of stage IIA, 11 cases of stage IIB, five cases of stage IIIA and five cases of stage IIIB.

### Transcatheter arterial chemoembolization

**Figure 1:**
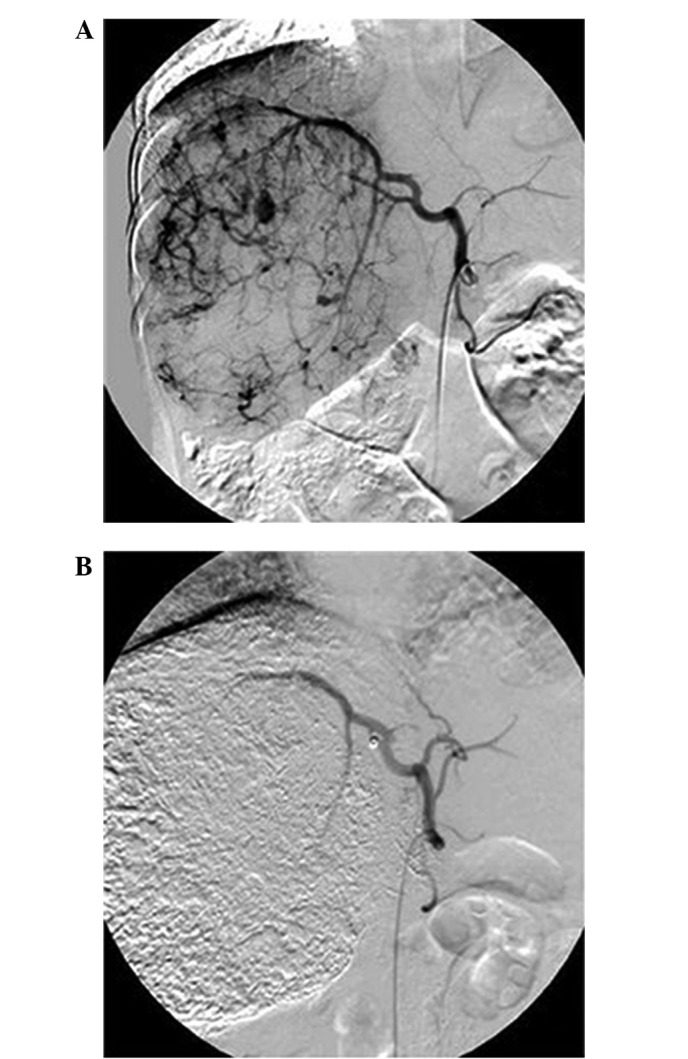
Hepatic angiography. (A) Pre-chemoembolization; hepatic arteriography shows the inhomogeneous hypervascular nature of the tumor. (B) Post-chemoembolization; lipiodol retained by the tumor after injection, outlining its large size.

Under general anesthesia and continued monitoring, including observations of ECG, blood pressure and oxygen saturation measurements, the femoral artery was cannulated using the Seldinger technique. A 4-Fr catheter was inserted through the femoral artery under fluoroscopy via digital subtraction angiography (DSA). A 2.7-Fr microcatheter was selectively inserted through the catheter into the tumor-feeding artery. Pirarubicin and cisplatin were used as anticancer agents. According to the volume of the tumor, pirarubicin (20–30 mg/m^2^) and cisplatin (50–60 mg/m^2^) emulsified in lipiodol were injected into the artery supplying the tumor, followed by superselective embolization using polyvinyl alcohol (PVA) until the blood flow was completely stagnated (Fig. 1). In order to achieve a tumor status that was acceptable for surgery, the TACE procedure was performed one to four times depending on the patient’s response. The interval between the last TACE and surgery was approximately six weeks.

### Post-treatment assessment

Four to six weeks after TACE, the tumor volume, as measured by abdominal CT, and the serum AFP level were regarded as indicators for judging the treatment outcome. The tumor volume was calculated by the following formula: Volume = 1/2 × (transverse diameter)^2^ × length. The tumor response was classified into four categories using the following criteria: Complete response (CR), complete disappearance of all tumors and normal level of AFP lasting more than four weeks; partial response (PR), a decrease of ≥50% in tumor volume and a significant reduction in AFP level, with no evidence of new lesions or progression in any lesion; non-response (NR), a decrease of <50% in tumor volume, with no evidence of new lesions; and progressive disease (PD), an increase of the tumor size by >25% or appearance of a new lesion.

To evaluate the toxicity of TACE, the alanine aminotransferase (ALT), creatinine (Cr) and creatine kinase (CK) levels of the 24 patients were tested prior to the first TACE and subsequent to the last TACE to assess liver, renal and cardiac function, respectively.

### Statistical analysis

All data were analyzed using SPSS software (version 11, SPSS Inc., Chicago, USA). The differences between pre- and post-TACE groups were evaluated by Student’s t-test. P<0.05 was considered to indicate a statistically significant difference.

## Results

### Tumor response to TACE

**Figure 2:**
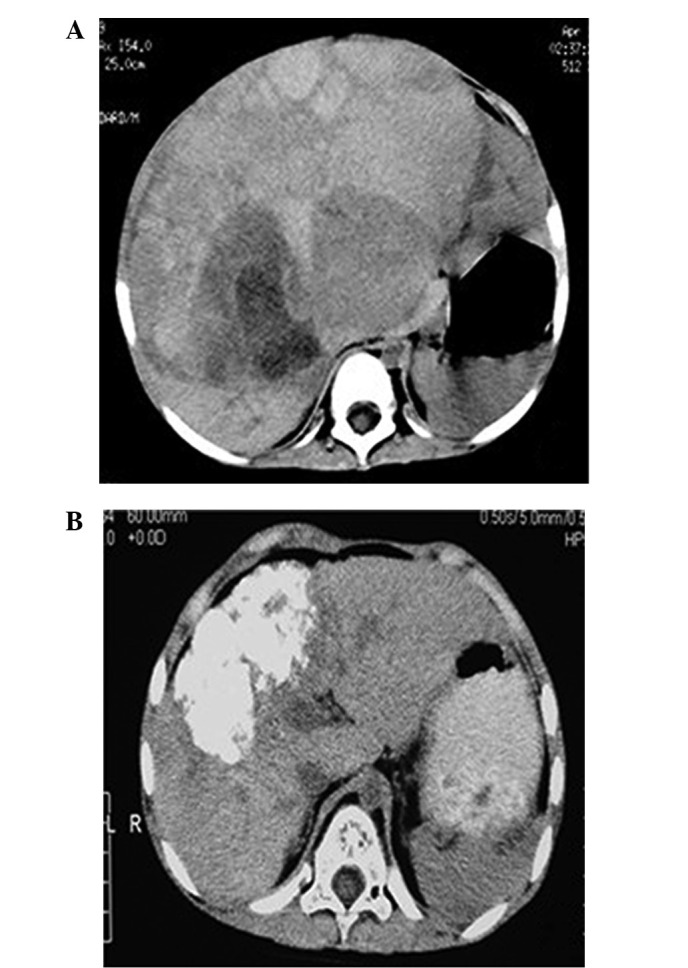
Non-contrast CT images of hepatoblastoma prior to the first TACE and following the last TACE. (A) Prior to the first TACE; CT image demonstrating a large tumor in the liver. (B) Following the last TACE; abundant lipiodol deposits appeared in the tumor, which was reduced significantly in volume. CT, computed tomography; TACE, transcatheter arterial chemoembolization.

Following TACE, there was a visible reduction in tumor size associated with a decrease in AFP levels (Table I). The responses of the tumors were favorable, as shown by the non-contrast abdominal CT (Fig. 2). The tumor volumes decreased by between 46.1 and 90.2%, with a mean value of 72%. The AFP levels fell by between 63.8 and 99.9%, with a mean value of 95.7%. According to the aforementioned criteria, there was a PR in 91.7% cases (22/24), although there were NRs in 8.3% cases (2/24). A total of 22 cases (PRs) underwent a subsequent safe complete surgical resection and the remaining two patients (NRs) accepted a partial resection. No delay to surgery was experienced.

### Adverse effects

Through statistical analysis, it was observed that no significant differences existed between the pre- and post-TACE groups with regard to Cr and CK. A significant difference was observed in the ALT levels (P<0.05), but this was not clinically significant as it was within the normal range (Table II). All patients presented with a fever following TACE and their temperatures ranged from 37.5 to 40ºC for two to 16 days. Other clinical symptoms included nausea and vomiting in 22 patients who recovered within three days following suitable treatment. No marked chemotherapeutic agent-induced toxicity was noted during TACE.

## Discussion

As previously mentioned, HB is one of the most common malignant tumors in children worldwide. Complete tumor resection is considered to be the most important treatment for the long-term survival of children with HB. However, approximately half of all children with HB have unresectable tumors at presentation due to a huge tumor size or extensive infiltration. Pre-operative systemic chemotherapy is vital for reducing tumor size and is able to convert an unresectable tumor to a resectable one (14,15). However, the systemic adverse effects may lead to a delayed surgery and even chemotherapy-related mortality (6,8,9,16). To reduce these disadvantages of systemic chemotherapy, an alternative targeting therapy is required.

TACE has been demonstrated to be a valuable approach and been used extensively in hepatocellular carcinoma (HCC) in adults (17,18). However, the application of TACE in children is limited as the setting and equipment must be adapted to infants and children. The small vessel diameter of pediatric vessels demands more experience and skill to avoid dissection or perforation. Pediatric TACE has become feasible with the development of the micro-catheter technique. In the present series, all of the cases showed a significant response to TACE, with a reduction in tumor size and a decrease in the AFP levels. The majority of cases underwent subsequent safe complete surgical resection and no delay to surgery was experienced. Angiography provides a comprehensive understanding of the nutrient vessels of the tumor, enabling subsequent intra-arterial injection with chemotherapy drugs and embolic agents. TACE exposes the tumor cells to high concentrations of drugs, which cannot be achieved by systemic administration using the same dose. For the purpose of chemoembolization, the ethiodized oil was conjugated with pirarubicin and cisplatin. The selectively accumulated emulsion releases the anticancer drugs slowly and decreases the transit time of the drugs through the tumor vasculature, thus resulting in an increased local concentration and tumor contact time for the drugs (16,19–21). As an effective embolic agent, PVA lead to the stenosis and occlusion of the artery supply to the tumor and its subsequent necrosis.

Although TACE is an outstanding method, there are certain complications that require attention. In the present study, a ‘post-embolization syndrome’ consisting of fever, abdominal pain, nausea, vomiting and elevated AST, ALT and CRP levels occurred in almost all patients, which may have been due to the massive necrosis of the tumor, although these symptoms were minimal and transient (6,22). More seriously, major complications associated with the use of chemoembolic agents, including acute liver failure, tumor rupture or pulmonary embolism, may also occasionally occur (22,23). In the present series, no marked chemotherapeutic agent-induced toxicity was observed during TACE. Serious adverse effects, including liver dysfunction, renal function failure, cardiac damage and myelosuppression, did not occur. Thus TACE may be considered a safe pre-operative treatment instead of systemic chemotherapy, particularly for patients without distant metastasis.

In conclusion, pre-operative TACE may be considered as an effective, feasible and safe treatment, instead of systemic chemotherapy, for inducing the surgical resectability of unresectable HB in pediatric patients during the surgical resection waiting period. However, further experience and research into its use is necessary prior to this approach becoming accepted as a first-line therapy.

## Figures and Tables

**Table I:** Therapeutic effects of TACE in 24 patients.

					Tumor volume (cm^3^)		AFP level (ng/ml)			
								
Case No.	Gender	Age	No. of TACEs	CCSG stage	Pre-TACE (first time)	Post-TACE (last time)	Pre-TACE (first time)	Post-TACE (last time)	Tumor shrinkage rate (%)	AFP decrease (%)
1	M	16 m	4	IIB	264.96	66.83	132002.00	374	74.78	99.72
2	M	13 m	3	IIB	685.45	160.62	484000.00	266.00	76.57	99.95
3	F	13 m	2	IIB	486.41	109.65	385462.00	4768.00	77.46	98.76
4	F	14 m	2	IIB	510.30	167.24	12568.00	9.55	67.23	99.92
5	F	13 m	4	IIIB	331.61	32.42	363000.00	45.80	90.22	99.99
6	F	19 m	1	IIIA	399.42	215.13	484000.00	175307.00	46.14	63.78
7	M	9 m	3	IIIA	329.16	106.02	34892.00	1023.00	67.79	97.07
8	M	22 m	2	IIIB	467.54	138.65	25066.00	262.00	70.34	98.95
9	M	13 m	2	IIA	740.70	90.32	127278.00	61.40	87.81	99.95
10	M	3 m	2	IIA	268.80	135.05	8920.00	453.00	49.76	94.92
11	M	1 m	4	IIB	375.56	87.72	52320.00	52.50	76.64	99.90
12	M	9 m	3	IIIB	305.92	78.46	24200.00	32.00	74.35	99.87
13	M	27 m	2	IIIB	330.75	124.93	12934.00	243.00	62.23	98.17
14	M	16 m	2	IIIA	700.05	206.54	4309.00	103.00	70.50	97.61
15	F	16 m	2	IIA	304.70	55.27	7074.00	4.78	81.86	99.93
16	M	26 d	3	IIB	205.22	72.86	10043.00	63.00	64.50	99.37
17	F	5 m	2	IIB	301.83	88.51	8652.00	31.10	70.68	99.64
18	F	9 m	3	IIB	575.09	89.21	20700.00	58.70	84.49	99.72
19	M	6 m	2	IIB	451.64	130.70	425226.00	1100.00	71.06	99.74
20	F	41 m	2	IIIA	518.64	175.73	294588.00	1203.00	66.12	99.59
21	F	37 m	2	IIIB	1466.45	268.35	310000.00	91800.00	81.70	70.39
22	F	10 m	1	IIIA	445.56	199.82	2390.00	340.00	55.15	85.77
23	M	5 m	2	IIB	336.73	58.14	1004.00	46.00	82.73	95.42
24	M	16 m	2	IIB	371.70	85.72	3658.00	15.00	76.94	99.59
Mean	-	13.91 m	-	-	465.48	122.62	134761.92	11569.24	71.96	95.73

TACE, transcatheter arterial chemoembolization; M, male; F, female; m, months; d, days; AFP, serum α-fetoprotein; CCSG, Children’s Cancer Study Group.

**Table II:** Toxicity of TACE.

Variable	Prior to first TACE	Following last TACE	P-value
ALT (U/l)	26.33±17.24	33.04±13.71	0.029
Cr (umol/l)	22.71±5.03	21.75±4.42	0.694
CK (U/l)	116.70±31.57	120.39±26.08	0.472

Variables are expressed as the mean ± SD and were compared using Student’s t-test. TACE, transcatheter arterial chemoembolization; ALT, alanine aminotransferase; Cr, creatinine; CK, creatine kinase.

## References

[1] Schnater JM, Kohler SE, Lamers WH (2003). Where do we stand with hepatoblastoma? A review. Cancer.

[2] Derek J, Perilongo G (2006). Hepatoblastoma: an oncological review. Pediatr Radiol.

[3] Czauderna P, Otte JB, Roebuck DJ (2006). Surgical treatment of hepatoblastoma in children. Pediatr Radiol.

[4] Nakagawa N, Cornelius AS, Kao SCS (1993). Transcatheter oily chemoembolization for unresectable malignant liver tumors in children. J Vasc Intervent Radiol.

[5] Arcement CM, Towbin RB, Meza MP (2000). Intrahepatic chemoembolization in unresectable pediatric liver malignancies. Pediatr Radiol.

[6] Oue T, Fukuzawa M, Kusafuka T (1998). Transcatheter arterial chemoembolization in the treatment of hepatoblastoma. J Pediatr Surg.

[7] Tashjian DB, Moriarty KP, Courtney RA (2002). Preoperative chemoembolization for unresectable hepatoblastoma. Pediatr Surg Int.

[8] Exelby PR, Filler RM, Grosfeld JL (1975). Liver tumors in children in the particular reference to hepatoblastoma and hepatocellular carcinoma: American Academy of Pediatrics surgical section survey - 1974. J Pediatr Surg.

[9] Evans AE, Land VJ, Newton WA (1982). Combination chemotherapy (vincristine, adriamycin, cyclophosphamide, and 5-fluorouracil) in the treatment of children with malignant hepatoma. Cancer.

[10] Vogl TJ, Scheller A, Jakob U (2006). Transarterial chemoembolization in the treatment of hepatoblastoma in children. Eur Radiol.

[11] Ohtsuka Y, Matsunaga T, Yoshida H (2004). Optimal strategy of preoperative transcatheter arterial chemoembolization for hepatoblastoma. Surg Today.

[12] Li JP, Chu JP, Yang JY (2008). Preoperative transcatheter selective arterial chemoembolization in treatment of unresectable hepatoblastoma in infants and children. Cardiovasc Intervent Radiol.

[13] Ortega JA, Krailo MD, Haas JE (1991). Effective treatment of unresectable or metastatic hepatoblastoma with cisplatin and continuous infusion doxorubicin chemotherapy: a report from the Children’s Cancer Study Group. J Clin Oncol.

[14] Seo T, Ando H, Watanabe Y (1998). Treatment of hepatoblastoma: less extensive hepatectomy after effective preoperative chemotherapy with cisplatin and adriamycin. Surgery.

[15] Ehrlich PF, Greenberg ML, Filler RM (1997). Improved long-term survival with preoperative chemotherapy for hepatoblastoma. J Pediatr Surg.

[16] Ogita S, Tokiwa K, Taniguchi H, Takahashi T (1987). Intraarterial chemotherapy with lipid contrast medium for hepatic malignancies in infants. Cancer.

[17] Groupe d’Etude et de Traitement du Carcinome Hépatocellulaire (1995). A comparison of lipiodol chemoembolization and conservative treatment for unresectable hepatocellular carcinoma. N Engl J Med.

[18] Llovet JM, Real MI, Montaña X (2002). Arterial embolization or chemoembolisation versus symptomatic treatment in patients with unresectable hepatocellular carcinoma: a randomized controlled trial. Lancet.

[19] Iwai K, Maeda H, Konno T (1984). Use of oily contrast medium for selective drug targeting to tumor: enhanced therapeutic effect and X-ray image. Cancer Res.

[20] Nakamura H, Hashimoto T, Oi H, Sawada S (1989). Transcatheter oily chemoembolization of hepatocellular carcinoma. Radiology.

[21] Sasaki Y, Imaoka S, Kasugai H (1987). A new approach to chemoembolization therapy for hepatoma using ethiodized oil, cisplatin, and gelatin sponge. Cancer.

[22] Sakamoto I, Aso N, Nagaoki K (1998). Complications associated with transcatheter arterial embolization for hepatic tumors. Radiographics.

[23] Chung JW, Park JH, Im JG (1993). Pulmonary oil embolism after transcatheter oily chemoembolization of hepatocellular carcinoma. Radiology.

